# Bactericidal Permeability Increasing Protein Deficiency Aggravates Acute Colitis in Mice by Increasing the Serum Levels of Lipopolysaccharide

**DOI:** 10.3389/fimmu.2020.614169

**Published:** 2021-01-21

**Authors:** Qingli Kong, Zhe Lv, Yun Kang, Yunqing An, Zhenlong Liu, Jianmin Zhang

**Affiliations:** ^1^ Department of Immunology, School of Basic Medical Sciences, Capital Medical University, Beijing, China; ^2^ Department of Research and Development, NVSI National Vaccine and Serum Institute, Beijing, China; ^3^ Department of Immunology, CAMS Key Laboratory for T cell and Immunotherapy, Institute of Basic Medical Sciences, Chinese Academy of Medical Sciences and School of Basic Medicine, Peking Union Medical College, State Key Laboratory of Medical Molecular Biology, Beijing, China

**Keywords:** bactericidal permeability-increasing protein, antibacterial peptide, ulcerative colitis, gram negative bacteria, gene knockout

## Abstract

**Objective:**

The objective of this study was to understand the role of bactericidal permeability increasing protein (BPI) in the pathogenesis of experimental murine colitis.

**Methods:**

We used the Cre-LoxP system to generate BPI knockout (BPI KO) mice. Acute colitis was induced in BPI KO mice and wild-type (WT) mice by subjecting the mice to 5% dextran sulfate sodium (DSS). Mice were observed for symptoms of experimental colitis. The survival of BPI KO mice to infection with *Acinetobacter baumannii*, a gram-negative bacterium, was also assessed.

**Results:**

Southern blot, RT-PCR, and western blot results showed that the 2^nd^ and 3^rd^ exons of the murine *Bpi* gene were knocked out systemically, confirming successful construction of the BPI KO mouse. BPI KO mice subjected to DSS showed increased symptoms of experimental colitis, increased colonic mucosal damage, increased epithelial permeability, elevated levels of serum LPS, and a disrupted fecal microbiome as compared with WT mice. Furthermore, BPI KO mice challenged intraperitoneally with *A. baumannii* died sooner than WT mice, and the total number of bacteria in the abdominal cavity, spleen, and liver was increased in BPI KO mice as compared to WT mice.

**Conclusions:**

We successfully generated BPI KO mice. The BPI KO mice developed worse colitis than WT mice by increased colitis symptoms and colonic mucosal damage, elevated levels of serum LPS, and a disrupted microbiome. BPI could be a potential target for treatment of ulcerative colitis in humans.

## Introduction

Bactericidal permeability-increasing protein (BPI), a cationic protein with a molecular weight of 55 kDa (1, 2), is found in the neutrophils of humans, cattle, pigs, mice, and other mammals that have bactericidal and neutralizing-endotoxins [e.g., bacterial lipopolysaccharide (LPS)] function (3, 4). In humans, BPI exists not only in neutrophils, eosinophils, and fibroblasts, but also in human mucosal epithelial cells, which are involved in the composition of the intestinal mucosal barrier (5, 6). The sequence of the murine *Bpi* gene was first reported in 2005. The mRNA expression of *Bpi* in nine tissues, including the heart, liver, spleen, lung, kidney, testis, and ovary, was measured, but positive expression was found only in the testis (7). Furthermore, the gene expression of BPI increased approximately 80–100 times in mouse neutrophils 24 h after the mice were injected with LPS (8). Our lab has prepared and identified murine *Bpi* gene systemic knockout plasmids (9) which can be used in this area of research.

Ulcerative colitis (UC) is one of two diseases that comprise IBD. UC is a chronic, non-specific colitis response, and its etiology is currently unknown (10). The manifestations of UC is diarrhea, abdominal pain, weight loss, and rectal bleeding. Histological, UC is limited to the mucosa by diffuse inflammatory cell infiltration of the mucosa with basal plasmacytosis, crypt architectural and a reduction of mucus-secreting goblet cells (11).

The stool composition and mucosal antibacterial response profile, especially BPI, have been shown to be related to the course of disease in patients with newly diagnosed UC. Additionally, the mucosal gene expression of BPI was found to be a good predictor of the disease course (12). The serum of patients with UC contains a variety of autoantibodies, such as antibodies against colonic epithelial cells, colon tissue, neutrophils, endothelial cells, heat shock proteins, and pancreatic proteins. Approximately 50–70% of patients with UC have been shown to have anti-neutrophil cytoplasmic autoantibodies (ANCAs) (11). The positive rate of BPI-ANCAs in UC increase with the severity of the patient’s condition (13, 14). In addition, there is genetic diversity at the 645^th^ base of the *Bpi*. Changing this base from A to G causes the 216^th^ amino acid of the BPI protein to change from a lysine (Lys) to glutamic acid (Glu). This mutation is associated with inflammatory bowel disease (15).

The purpose of this study was to investigate of the role of BPI in experimental colitis. Here, we constructed BPI-floxed and BPI knockout (BPI KO) mice. We show that BPI KO mice had reduced antibacterial function. Furthermore, we subjected wild-type (WT) mice and BPI KO mice to 5% dextran sulfate sodium and assessed the development of experimental colitis in these mice.

## Materials and Methods

### Mice

B6Ei.129S4-Tg (Prm-cre)58Og/EiJ.129 mice (stock #007252) were purchased from the Jackson Laboratory (Bar Harbor, ME, USA) (16). The BPI floxed 129 mouse strain and the BPI knockout 129 strain mouse strain were bred at the Experimental Animal Center of Capital Medical University (Beijing, China). All animal experiments were approved by the Animal Experiment and Experimental Animal Welfare Committee of Capital Medical University (approval numbers: AEEI-2016-010 and AEEI-2019-012, respectively). The above mice and WT (129 strain) mice were housed in micro-isolated cages with filtered air under sterile conditions, free access to sterile water and autoclaved food. The mice were 8–10 weeks old and weighed 20–30 g for all experiments. During the experiment, 0.2ml pentobarbital (0.8%) was injected intraperitoneally near the time of death to alleviate the pain of the mice.

### Generation of Bactericidal Permeability Increasing Protein Knockout Mice

We used the Cre/LoxP system to insert LoxP sites on both sides of the 2^nd^ and 3^rd^ exons of the mouse (C57BL/6J) *Bpi* gene in order to obtain the BPI conditional gene-targeting (BpiKO) vector (17). The BpiKO vector was transfected into mouse (129 strain) embryonic stem (ES) cells. After introducing the target ES cells into the blastocyst cavity of the recipient C57BL/6J mice by microinjection, the blastocysts were transplanted into pseudopregnant female mice to obtain BPI floxed heterozygous mice (BPI^fl/-^). The BPI^fl/-^ mice (on a 129 background) were mated with Prm-cre mice, which yielded the F1 generation of BPI KO heterozygous (BPI^+/-^) mice. BPI^+/-^ mice were then mated to obtain BPI KO homozygous (BPI^-/-^, or BPI KO) mice. BPI floxed mice and BPI KO mice were bred for more than 10 generations. The BPI KO did not affect the reproduction of offspring, and BPI KO mice did not present with any disease.

### Identification of Bactericidal Permeability Increasing Protein Knockout Mice

#### Southern Blot and PCR

DNA was extracted from mouse tails and digested with the restriction enzyme NcoI. The product was separated by electrophoresis and transferred to a nylon membrane. Digoxin-labelled probes prox-3f: ATCTCCACCGACCTGATTCT and prox-3r: GGCAGATGGCGTAAGAGCAT were used to hybridize to the DNA.

#### PCR Identification

DNA from tail or ear of mice was extracted, and primers were designed outside of the LoxP1/2 loci at both ends of the 2^nd^ and 3^rd^ *Bpi* gene exons. The target gene was amplified by PCR, and 1.5% gel electrophoresis and sequencing were performed.

#### RT-PCR Identification

Mice were injected with 200 ng LPS intraperitoneal injection (i.p.). Twenty-four hours post-injection, RNA was extracted from both the testis tissue and neutrophils isolated from the bone marrow and spleen of mice by density gradient centrifugation and lysis of erythrocytes (Solabio, Beijing, China) as reported previously (18). RNA was extracted from the colonic mucosa after the mice were subjected to 5% DSS for 5 days. One microgram of tissue-derived RNA was reverse transcribed into 20 µL of cDNA, and 1 µL of the cDNA was used for amplifying the BPI fragment with primer P1 (5’-AACGTGCGGAAATGGTCAG-3’) and primer P2 (5’-CAGTTGGAGCAGATGGTGGT-3’). The products were confirmed by 1.5% gel electrophoresis and sequenced by Sangon Biotech Co., Ltd (Shanghai, China). The murine β-actin gene was amplified using the following primers: 5’-TGGAATCCTGTGGCATCC A-3’ and 5’-TAACAGTCCGCCTAGAAGCAG-3’.

#### Western Blot

Splenic neutrophils were isolated 24 h post-injection of 200 ng LPS by density gradient centrifugation (Solabio, Beijing, China) as reported previously (18). Mouse splenic neutrophils and testis tissue were lysed in RIPA buffer (Applygen Technology Inc., Beijing, China) with protease inhibitors (Applygen Technology Inc., Beijing, China). The protein lysate was analyzed *via* western blot to confirm that BPI was knocked out. The plasmid pET28a-muBPI36-259 was previously prepared in 2008 (17). The plasmid was delivered to Gegen Biotechnology Co., Ltd. (Beijing, China), and the mouse monoclonal antibody (IgG) against mouse BPI amino acids 36–259 was prepared by this company, which used as the primary antibody(1:1000 dilution) in western blot. The secondary antibody was a fluorescent antibody against mouse IgG (IRDye 800CW goat anti-mouse, cat #: 926-32210; LI-COR, Lincoln, Nebraska USA) (1:10,000 dilution). The Odyssey Sa Infrared Imaging System (LI-COR, Lincoln, Nebraska USA) was used to visualize the western blots.

#### Routine Blood Test

Retro-orbital blood (200 µl) was obtained from mice, placed in a tube with EDTAK2(SANLI China, Liuyang, Hunan, China) to prevent coagulation, and transported at 4°C to the Institute of Experimental Animals, Chinese Academy of Medical Sciences(Beijing, China) where the blood was analyzed. The instrument used for the blood cell differential counts was ABX-DX-120 (Horiba Medical, Montpellier, France).

### Resistance of Mice to Lethal Doses of Bacterial Infection

Mouse survival was observed after a lethal dose of *Acinetobacter baumannii* (ATCC: BAA-2469). *A. baumannii* was cultured in a Nutrient Broth (Cat 234000; BD, Shanghai, China) in a 37°C incubator for 12 to 16 h. The bacteria were diluted with physiological saline with 5% inactivated yeast(ANGEL YEAST CO.,LTD, Yichang, Hubei, China) to a concentration of 1 × 10 (4) Colony-Forming Units(CFU)/ml. Each mouse was i.p. injected with 0.5 ml of the bacterial suspension. Survival of the mice was observed for 72 h post-infection, and all deaths were recorded (19).

Furthermore, to check the multiplication of bacteria for the cause of the death, *A. baumannii* challenge was diluted to 1 × 10^4^ CFU/ml with 5% inactivated yeast. Each mouse was injected i.p. with 0.5 ml of the solution. At 16 h post-injection, the abdominal cavity was washed with 1.5 ml of sterile physiological saline and diluted from 1 × 10^−3^ to 1 × 10^−5^. The liver and spleen were also collected to count the bacteria. After rinsing with physiological saline, the organs were put in a tube with 10 ml of physiological saline, homogenized with a super-speed tissue homogenizer (IKA, T18; IKA^®^-Werke GmbH & Co., Staufen, Germany) at 13,000 × g, and then the homogenate was diluted to 1 × 10^−4^ with physiological saline. Bacterial counts were obtained using Pour Plate Method (from https://www.bd.com). After centrifuging the remaining peritoneal fluid at 250 × g, the solution was resuspended with normal saline to count the cells in the peritoneal cavity. Twenty microliters of peritoneal fluid were collected on a glass slide. After Wright staining, phagocytes were counted using a microscope (NIKON, Ci-s; Yokohama, Kanagama, Japan) at 400 times magnification.

### Dextran Sulfate Sodium-Induced Colitis Model in Mice

The mice were given 5% DSS (DSS MW: 36,000–50,000; MP Biomedicals United States, Solon, OH USA) in their drinking water for 5 days to induce a model of acute experimental colitis as reported previously (20). The mice were observed daily, and the weight, stool consistency, and presence of blood in the stool were recorded. On the 5^th^ days drinking 5% DSS, the mice were sacrificed and the proximal end of colon was collected. The paraffin sections of proximal end of colon were prepared. Hematoxylin and eosin (H&E) and Alcian blue-periodic acid sthiff (AB-PAS) staining were used to observe the pathological damage and inflammatory cell infiltration into the colon.

Based on previously reported methods (20), During the duration of the experiment, a disease activity index (DAI) score can be assessed to evaluate the clinical progression of colitis. The DAI is the combined score of weight loss compared to initial weight, stool consistency, and bleeding. Scores are defined as follows: weight loss: 0 (no loss), 1 (1–5%), 2 (5–10%), 3 (10–20%), and 4 (>20%); stool consistency: 0 (normal), 2 (loose stool), and 4 (diarrhea); and bleeding: 0 (no blood), 1 (Hemoccult positive), 2 (Hemoccult positive and visual pellet bleeding), and 4 (gross bleeding, blood around anus). DAI can be scored daily during the duration of the DSS treatment.

Colonic pathology scores for each group were obtained as reported previously (20). H&E stained colonic tissue sections are scored by a blinded observer using a previously published system for the following measures: crypt architecture (normal, 0—severe crypt distortion with loss of entire crypts, 3), degree of inflammatory cell infiltration (normal, 0—dense inflammatory infiltrate, 3), muscle thickening (base of crypt sits on the muscularis mucosae, 0—marked muscle thickening present, 3), goblet cell depletion (absent, 0—present, 1) and crypt abscess (absent, 0—present, 1).

### Immunofluorescence Microscopy

After the mice were subjected to 5% DSS for 5 days, the paraffin sections of the proximal end of colon were prepared. The colon sections were deparaffinized in xylene and rehydrated through graded ethanol. Antigen retrieval was performed in citrate buffer with 750-W microwave for 20 min. Endogenous peroxidase was blocked using 3% H_2_O_2_ for 1h. Nonspecific sites were blocked by using 3% BSA for 1h. The sections were incubated with mouse monoclonal IgG (Gegen Biotechnology Co., Ltd. Beijing, China) against mouse BPI amino acids 36–259, which used as the primary antibody (1:200 dilution) over night at 4°C. Then the sections were incubated with Goat anti mouse IgG (FITC labeled) (Applygen Technology Inc., Beijing, China) (1:4,000 dilution) for 1h at room temperature. At last, the slides were covered with the DAPI mounting medium (Solarbio, Beijing, China), observe with fluorescence microscope (Leica DM6000B, German) and digitally captured images. Finally, the slides were mounted with the DAPI mounting medium (Solarbio, Beijing, China) to mount slides. Slides were visualized with the fluorescence microscope (Olympus, BX61).

### Measurement of Epithelial Permeability *In Vivo*

As described previously, (21) we used blood concentration of FITC–dextran (MW 3.0–5.0 kDa, Sigma, USA) to measure colonic epithelial permeability. The mice were given 5% DSS for 5 days. Before experiments, mice were fasted for 15h, but had unlimited access to 5% DSS. At 5^th^ day, mice were administered by gavage 0.6 mg/g body weight FITC–dextran (1×PBS containing 30 mg/ml FITC–dextran). After 4h, the mice were anaesthetized, and Retro-orbital blood was sampled into heparinized tubes. Plasma concentration of fluorescein was measured using a microplate spectrofluorimeter (SYNERGY, BIO Tek Instruments, Inc, Winooski, USA) with excitation wavelength of 485 nm and an emission wavelength of 520 nm, serially diluted FITC–dextran as standard.

### Analysis of Serum Proteins

After the mice were subjected to 5% DSS for 5 days, the peripheral blood of mice was isolated, and the serum was collected. The LPS level in the serum was determined by a limulus amebocyte lysate-based assay (Xiamen Bioendo Reagents Experimental Factory Co., Ltd.Xiamen, China). An LPS Binding protein (LBP) ELISA kit (Hycult, Uden, Netherland) was used to detect the LBP levels in mouse serum.

### Detection of Myeloperoxidase and Cytokine Levels

After the mice were subjected to 5% DSS for 5 days, 1 cm of the mouse proximal end of colon was added to 0.5 mL of tissue lysate (RIPA lysate and protease inhibitor; Applygen Technology Inc., Beijing, China). After homogenization and centrifugation at 12,000 × g, the supernatant was collected, and the total protein concentration was measured by BCA (Applygen Technology Inc., Beijing, China). Myeloperoxidase (MPO) levels were detected using an MPO ELISA kit (RayBiotech, Guangzhou, China), and cytokine levels were detected using a protein chip (QAM-CYT-1-1, RayBiotech, Guangzhou, China).

### 16S rDNA Analysis in Mouse Stool Samples

Mouse feces was collected and stored at −80°C. The samples were then sent to Sangon Biotech Co., Ltd (Shanghai, China) for detection of the bacterial flora in the stool samples. E.Z.N.ATM Mag-Bind Soil DNA Kit (OMEGA, Shanghai, China) was used to extract DNA from mouse feces. Primer 341F: CCCTACACGACGCTCTTCCGATCTG (barcode) CCTACGGGNGGCWGCAG and primer 805R: GACTGGAGTTCCTTGGCACCCGAGAATTCCAGACTACHVGGGTATCTAATCC were used to amplify V3–V4 region of DNA of the bacterial flora. Then sequencing and data analysis were finished by Sangon Biotech Co., Ltd (Shanghai, China).

### Statistical Analysis

All data are expressed as the mean ± standard deviation (SD) and were analyzed using GraphPad Prism, version 6.01 (San Diego, CA, USA). Statistical comparisons were performed using an unpaired *t* test and Chi squared test. * *P* < 0.05, ** *P* < 0.01, and *** *P* < 0.001 were considered statistically significant.

## Results

### Generation of Bactericidal Permeability Increasing Protein Knockout Mice

**Figure 1:**
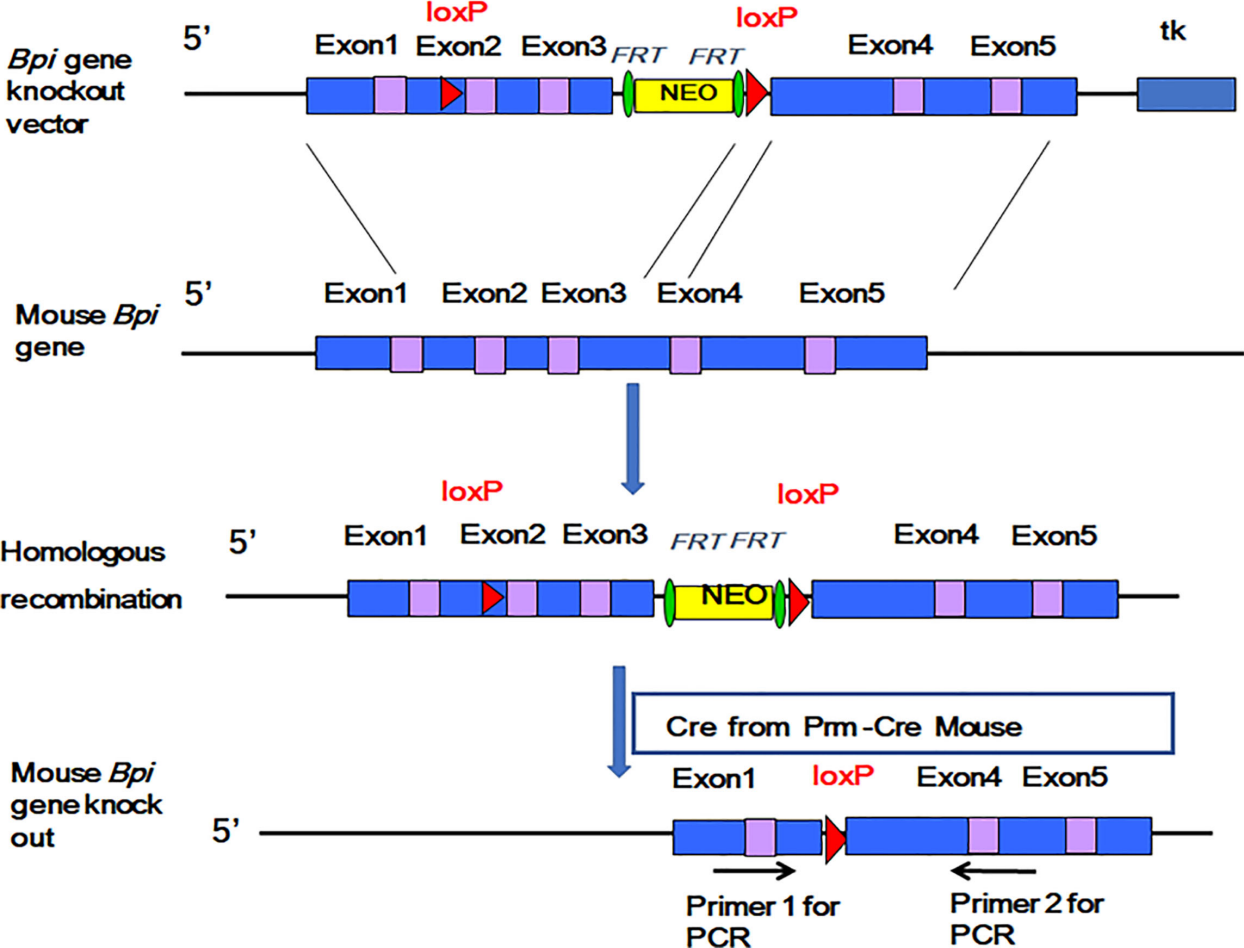
A diagram of the construction of the BPI knockout mouse.

We inserted LoxP sites on both sides of the 2^nd^ and 3^rd^ exons of the mouse *Bpi* gene to obtain the BpiKO vector (**Figure 1**, top). The BpIKO vector was transfected into ES cells. The target ES cells were then transplanted into pseudopregnant female mice to obtain BPI^fl/-^ mice (**Figure 1**, middle). BPI^fl/-^ mice on a 129 background were mated with Prm-cre mice, and the F1 generation of BPI^+/-^ mice was obtained (**Figure 1**, bottom).

### Identification of Bactericidal Permeability Increasing Protein Knockout Mice

**Figure 2:**
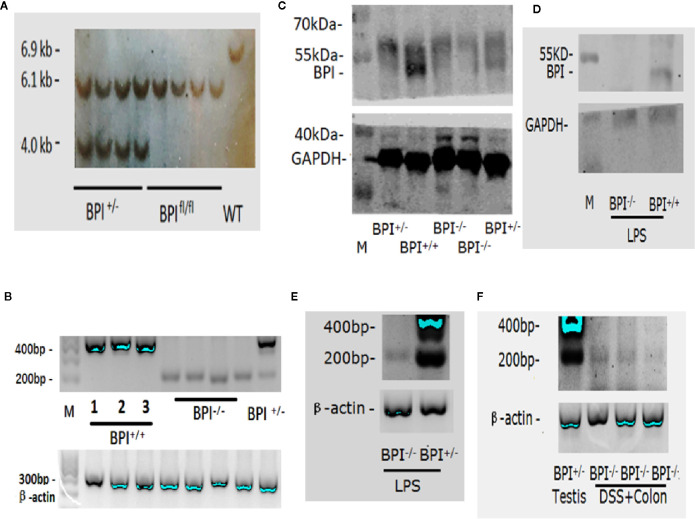
Identification of BPI knockout mice. **(A)** Southern blot analysis of BPI KO heterozygous (BPI^+/-^), BPI floxed homozygous (BPI^fl/fl^), and WT mice. WT mice had a band at 6.9 kb, BPI^fl/fl^ mice had a band at 6.1 kb, and BPI^+/-^ mice had a band at 6.1 kb and at 4 kb. **(B)** RT-PCR identification of mouse testis tissue. BPI^+/+^ mice (including WT and BPI^fl/fl^) showed a band at 400 bp. BPI KO homozygous mice (BPI^-/-^) mice showed a band at 200 bp. BPI KO heterozygous (BPI^+/-^) mice showed both a 400 bp band and a 200 bp band. **(C)** Western blot Identification of testis tissues. BPI^+/+^ mice showed a strong target band at 55 kDa. BPI^+/-^ mice showed a weak target band at 55 kDa. BPI^-/-^ mice did not show a band at 55 kDa. **(D)** Western blot identification of splenic neutrophils. After LPS stimulation, BPI^+/+^ mice showed a target band at 55 kDa, while BPI^-/-^ mice did not show a band. **(E)** After LPS stimulation, RNA from murine bone marrow-derived neutrophils was extracted and amplified by RT-PCR. BPI^-/-^ mice showed a band at 200 bp, and BPI^+/-^ mice showed a band at 400 bp and 200 bp. **(F)** After mice were subjected to 5% DSS for 5 days, RNA was obtained from the colonic epithelial tissue from BPI^-/-^ mice and amplified by RT-PCR. A 200-bp band was amplified. RNA from BPI^+/-^ mouse testis was used as a control.

Southern blot was performed to confirm the genomic deletion in the BPI KO mice. As expected, the results showed a band in WT mice at 6.9 kb, a band in BPI^fl/fl^ mice at 6.1 kb, and two bands, 6.1 kb and 4 kb, in BPI^+/-^ mice (**Figure 2A**).

In 2005, it was reported that mouse testis highly expressed BPI by Northern blot (7). Our RT-PCR results confirmed that the testis of BPI^+/+^ mice (including WT and BPI^fl/fl^ mice) highly expressed gene fragments at approximately 400 bp. The sequencing results confirmed the expression of mRNA fragments from the 1^st^ to 4^th^ exons, while the BPI KO mouse expressed gene fragments at approximately 200 bp (**Figure 2B**), which was confirmed by our sequencing results to be the remaining mRNA fragment after deletion of the 2^nd^ and 3^rd^ exons (see online **Supplementary Figure 1**). Our Western blot results confirmed that the BPI^+/+^ mouse showed strong target bands at approximately 55 kDa, and BPI^+/-^ mouse testis showed weak target bands at approximately 55 kDa. The BPI^-/-^ mouse had no 55 kDa band (**Figure 2C**).

In 2006, it was reported that the neutrophils of mice express BPI after LPS stimulation (13). Thus, we used LPS to stimulate BPI^-/-^ and BPI^+/-^ mice for 24 h. RT-PCR results showed that bone marrow-derived neutrophil precursor cells from LPS-treated BPI^-/-^ showed the 200bp band, whereas BPI^+/-^ mice showed the 200bp band and 400bp band (**Figure 2E**). However, BPI was not detected in the bone marrow-derived neutrophils by western blot.

Consequently, we stimulated mice with LPS for 24 h, and then isolated murine splenic neutrophils and performed western blot. The results confirmed that BPI^+/+^ mice showed a band at approximately 55 kDa and BPI^-/-^ mice did not show a 55 kDa band (**Figure 2D**).

In 2017, it was reported that BPI can be expressed in the intestine of mice infected with *Salmonella typhimurium* (22). As such, we extracted RNA from the colon epithelial tissue of mice undergoing experimental colitis *via* 5% DSS. The cDNA was set-up in a RT-PCR reaction with *Bpi* primers. The amplified product was resolved on a gel and resolved at the expected 200bp size band from BPI^-/-^(BPI KO) mice (**Figure 2F**).

### Neutrophil Number Was Not Affected in Bactericidal Permeability Increasing Protein KnockoutMice

**Figure 3:**
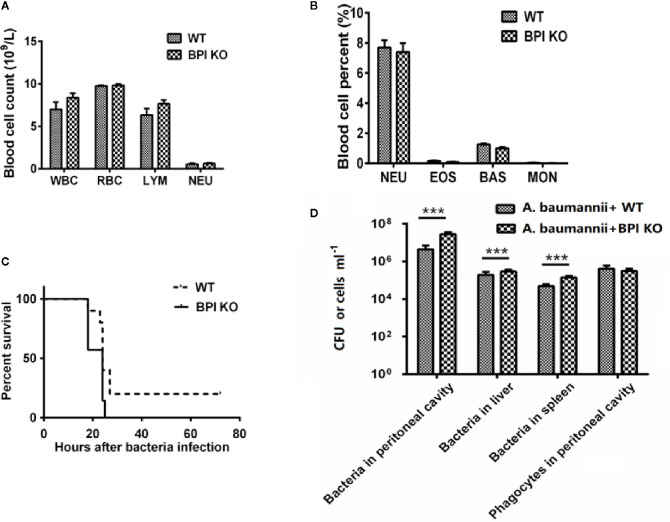
Survival of mice after intraperitoneal bacterial challenge. **(A, B)** the mice were used to count blood cells, the above values were mean ± standard deviation, BPI KO mice (n = 4), WT mice (n = 4). The name of abbreviation in the figure **(A, B)**: White blood cells (WBC), Red blood cells (RBC), Lymphocytes (LYM), Neutrophils (NEU), Eosinophils (EOS), Basophils (BAS), Monocytes (MON). **(C)** Survival of mice 72 h after an i.p. injection of *Acinetobacter baumannii* (0.5 × 10^4^ CFU) in 5% inactivated yeast solution. BPI KO mice (n = 10), WT mice (n = 10). **(D)** The number of bacteria and peritoneal phagocytes in the mice after intraperitoneal challenge with *A. baumannii*. BPI KO mice (n = 5), WT mice (n = 5), ***p < 0.001. All experiments were repeated two times independently.

WT mice and BPI KO mice underwent blood cell count. The results showed that WT mice and BPI KO mice had no statistically significant differences in the number of white blood cells, red blood cells, or neutrophils (**Figures 3A, B**).

### Resistance of Mice to Lethal Doses of Bacterial Attack

In 2013, it was reported that *A. baumannii* was sensitive to BPI (23). Thus, we challenged mice with *A. baumannii* to confirm the loss of BPI function. When challenged with a lethal dose of *A. baumannii*, all BPI KOmice died (n = 10) within 72 h after injection, while 20% of the WT mice (n = 10) survived longer than 72 h (**Figure 3C**). This indicated that KO mice were more vulnerable to challenge with *A. baumannii*. After *A. baumannii* i.p. injection, the number of bacteria in the peritoneal lavage fluid of BPI KO mice was significantly higher than that of WT mice (*P* < 0.001, n = 5). The bacterial counts in the spleen and liver homogenate of BPI KO mice were significantly higher than that in WT mice (*P* < 0.001, n = 5). BPI KO mice had more peritoneal phagocytes than WT mice, but the difference was not significant (**Figure 3D**), indicating that the decreased resistance to *A. baumannii* in the BPI KO mice was not due to a reduction in phagocytes (including neutrophils).

### Knockout of Bactericidal Permeability Increasing Protein Aggravated Dextran Sulfate Sodium-Induced Murine Colitis

**Figure 4:**
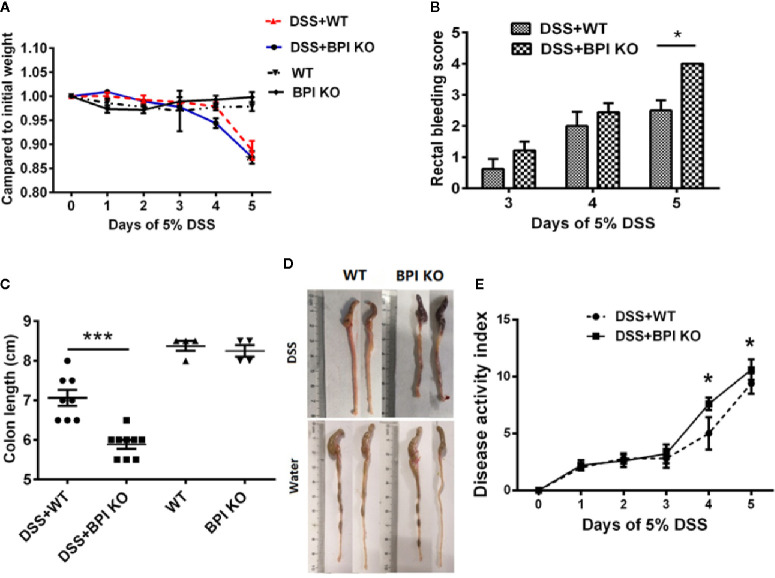
BPI deficiency aggravates colitis symptoms of DSS-induced murine colitis. WT mice (n = 5–8) and BPI KO mice (n = 5–9) were subjected to 5% DSS for 5 days. WT mice (n = 4) and BPI KO mice (n = 4) continuously drank water for 5 days as control. On the 5^th^ day, all mice were sacrificed. **(A)** During the 5-day observation period, changes in body weight were recorded and are expressed as a percentage of the body weight at the start of the experiment. **(B)** Bloody stool scores, *p < 0.05. **(C)** Length of the large intestine, ***p < 0.001. **(D)** General morphology of proximal end of colon on the 5^th^ day after DSS induction. **(E)** Disease activity index (DAI), *p < 0.05, ***p < 0.001. All experiments were performed three times.

We used 5% DSS to induce experimental colitis in mice. Both BPI KO and WT mice began to lose weight on the 3^rd^ day after exposure to 5% DSS in their drinking water. The decrease in weight was the most obvious on the 5^th^ day, which was significantly lower than that of control mice given regular water. When comparing the two groups of mice subjected to 5% DSS, we found that although BPI KO mice lost more weight than the WT mice, there was no significant difference between the two groups (**Figure 4A**). After three days of drinking 5% DSS, the stool of both BPI KO and WT mice was positive for rectal occult blood. The bleeding symptom was aggravated on the 4^th^ day and aggravated on the 5^th^ day. There was a significant difference between the two groups on the 5^th^ day (*P* = 0.039) (**Figure 4B**). BPI KO mice and WT mice given regular drinking water were negative for rectal occult blood (data not shown). After 5 days of drinking 5% DSS, the BPI KO mice had significantly shorter colons as compared to the WT mice (*P* = 0.004) (**Figures 4C, D**). The BPI KO mice had significantly higher DAI than the WT mice (P = 0.0147) at 4^th^ day, and (P = 0.0005) at 5^th^ day (**Figure 4E**).

**Figure 5:**
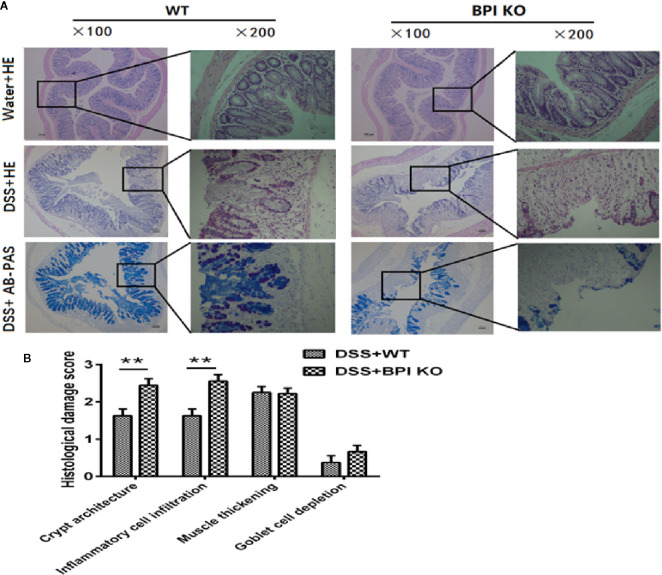
BPI deficiency aggravates colonic mucosal damage of DSS-induced murine colitis. WT mice (n = 5) and BPI KO mice (n = 5) were subjected to 5% DSS for 5 days. WT mice (n = 4) and BPI KO mice (n = 4) continuously drank water for 5 days as control. On the 5^th^ day, all mice were sacrificed. **(A)** Colonic images after H&E staining and AB-PAS staining (original magnification x 100 and x 200). The difference between the two groups was mainly within the colonic mucosa, such as severe crypt distortion, inflammatory cell infiltration and globlet cell depletion. **(B)** Colonic pathological scores, **p < 0.01. All experiments were performed three times.

After 5 days of drinking 5% DSS, H&E and AB-PAS staining of the proximal end of colon revealed that the damage scores for the BPI KO mice were higher than those of the WT mice, with significant differences in crypt curves (*P* = 0.0056) and inflammatory cell infiltration (*P* = 0.0023). The difference between the two groups was mainly within the colonic mucosa (**Figures 5A, B**). Taken together, these results suggest that a deficiency in BPI aggravates acute experimental colitis in mice.

### Localization of Bactericidal Permeability Increasing Protein Protein to the Crypt of Colon

**Figure 6:**
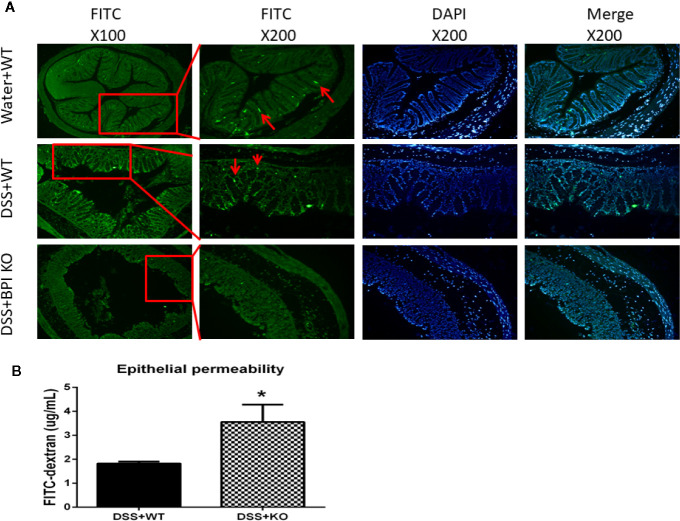
BPI KO leads to an increase of epithelial permeability of colon of DSS-induced mouse colitis. WT mice (n = 3) and BPI KO mice (n = 3) were subjected to 5% DSS for 5 days. WT mice (n = 3) continuously drank water for 5 days as control. **(A)** Under the fluorescence microscope, WT mice expressed BPI dominantly at sidewall and bottom of crypt of colon visualized by FITC fluorescence (green). In colitis model, WT mice expressed BPI dominantly at bottom of crypt and BPI deficiency was at colon of BPI KO mice. **(B)** Plasma concentration of fluorescein was measured at 4h after gavage FITC–dextran, *p < 0.05. All experiments were performed two times.

Next, we performed immunofluorescence to examine the localization of BPI. Photographs with the fluorescence microscope showed that BPI was predominantly expressed at sidewall and bottom of colon crypts in WT mice and the signal was weak. In colitis model, the level of BPI was increased at bottom of colon crypts but decreased at the sidewall of crypt which BPI deficiency was at colon of BPI KO mice (**Figure 6A**).

### Bactericidal Permeability Increasing Protein Knockout Leads to an Increase in Epithelial Permeability of Colon of Dextran Sulfate Sodium-Induced Murine Colitis

Then we sought to examine whether BPI deficiency affects the intestinal epithelial permeability. On the 5^th^ day of 5% DSS drinking, plasma concentration of fluorescein was measured at 4h after gavage FITC–dextran. The plasma FITC-dextran levels in the BPI KO mice (3.561 ± 0.4185, n = 3) were significantly higher than those in the WT mice (1.825 ± 0.04794, n = 3) (*P* = 0.0146) (**Figure 6B**). These results suggest that loss of BPI results in an increase of the epithelial permeability in colon, which might cause LPS release from lumen of colon into blood.

### Increased Lipopolysaccharide in the Serum of Bactericidal Permeability Increasing Protein Knockout Mice Leads to Aggravation of Dextran Sulfate Sodium-Induced Mouse Colitis

**Figure 7:**
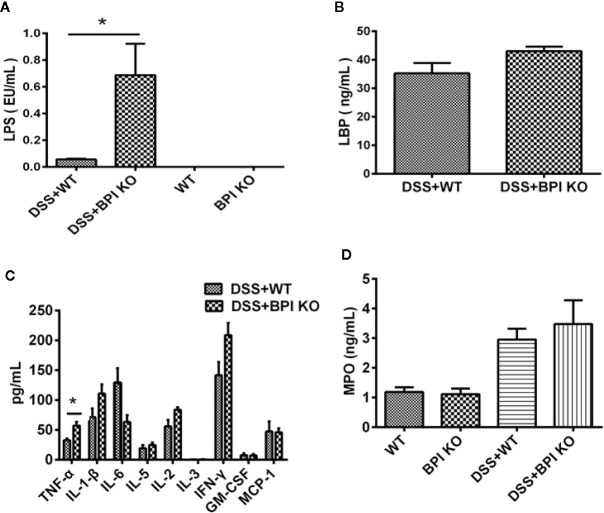
BPI deficiency increases serum LPS levels of DSS-induced murine colitis. WT mice (n = 5) and BPI KO mice (n = 5) were subjected to 5% DSS for 5 days. WT mice (n = 4) and BPI KO mice (n = 4) continuously drank water for 5 days as control. On the 5^th^ day, all mice were sacrificed. **(A)** Serum LPS levels s, * *p* < 0.05. **(B)** Serum LBP levels. **(C)** Cytokines levels in proximal end of colon tissues, * *p* < 0.05. **(D)** MPO levels in proximal end of colon tissue. All experiments were performed three times.

On the 5^th^ day of 5% DSS drinking, we did not detect any bacteria in the anti-coagulated blood and splenic homogenates collected from the mice (data not provided). Therefore, we measured the LPS levels in the serum of the mice. The serum LPS levels in the BPI KO mice were significantly higher than those in the WT mice (*P* =0.0159) (**Figure 7A**). Five days after the mice started drinking 5% DSS, the serum LBP levels in the BPI KO mice (n = 5) were higher than those in the WT mice (n = 5), but the difference was not significant (**Figure 7B**). Our results suggest that loss of BPI can increase the levels of LPS in the blood.

A cytokine assay using the colonic tissue of mice subjected to 5% DSS for 5 days revealed that the levels of TNF-α, IL-1β, IL-2, IL-3, and IFN-γ were higher ​​in BPI KO mice than in WT mice, while the levels of IL-6, GM-CSF, and MCP-1 were lower than those of the BPI KO mice (**Figure 7C**). Only the difference in the TNF-α levels was significant between the BPI KO mice and WT mice (*P* = 0.0267).

MPO detection in mouse colon tissue found that BPI KO mice had higher MPO values ​​than WT mice on the 5^th^ day of 5% DSS drinking, but the difference was not significant (**Figure 7D**).

### 16S rDNA Analysis in Mouse Stool Samples

**Figure 8:**
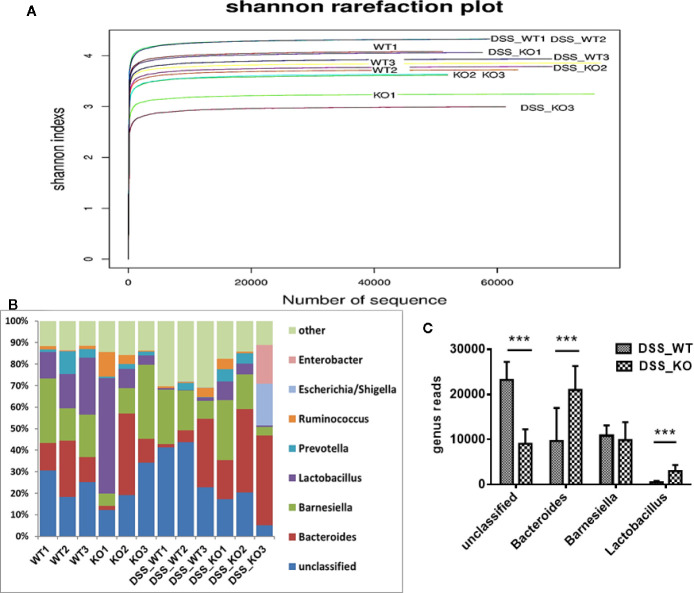
16S rDNA analysis in mouse stool samples. **(A)** Shannon rarefaction plot. The Shannon rarefaction plot showed that the microbial diversity of the feces from DSS_KO (DSS+ BPI KO) mice was lower than that of the DSS_WT (DSS+WT) mice. It was demonstrated that BPI knockout could decreased the microbial diversity of the feces. **(B, C)** Heatmap of the genera. The number of unclassified, *Bacteroides*, *Barnesiella*, and *Lactobacillus* as compared to the total number of bacteria in the DSS_WT and DSS_KO mice, ***p < 0.001. All experiments were performed twice.

We collected feces from the BPI KO mice to assess microbial differences by high-throughput 16S ribosomal DNA gene sequencing. The Shannon rarefaction plot demonstrated that the microbial diversity of the feces from KO (BPI KO) mice was lower than that of the WT mice. The Shannon rarefaction plot further demonstrated that the microbial diversity of the feces from DSS_KO (DSS+ BPI KO) mice was lower than that of the DSS_WT (DSS+WT) mice (**Figure 8A**). It was demonstrated that BPI knockout could decreased the microbial diversity of the feces. The heatmap of the genera shows the differences between the various species of flora in the DSS_WT and DSS_KO mice. The greatest differences in the number of bacteria between the DSS_KO and the DSS_WT groups were in unclassified (*P* < 0.0001, *X* ^2^ = 68951, n = 3), *Bacteroides* (*P* < 0.0001, *X* ^2^ = 14750, n = 3), and *Lactobacillus* (*P* < 0.0001, *X* ^2^ = 5177, n = 3) (**Figures 8B, C**). Taken together, these results suggest that a deficiency in BPI regulate the fecal flora in the gut.

## Discussion

BPI plays an important role in innate immunity against infection. The inability to produce BPI in newborns is the reason for their susceptibility to gram-negative infections (24). Human BPI is mainly expressed in neutrophils, where it has a bactericidal function, (25) and it has also been demonstrated in neutrophil extracellular traps (26). In this study, we generated BPI global knockout mice, and challenged mice with *A. baumannii*, a gram-negative bacterium (23). Our results showed that the number of *A. baumannii* in the peritoneal, liver and spleen was increased in BPI KO mice. It may be the lost the bactericidal function of BPI in BPI KO mice. Our previous studies showed that the serum LPS levels in mice challenged with *E. coli O111B4*, a gram-negative bacterium increased as the number of bacteria in the liver and spleen increased (27).

BPI is expressed in intestinal epithelial cells, which is the first barrier against infection. Human gastrointestinal epithelial cells also express BPI (6). Both BPI floxed mice and BPI KO mice did not spontaneously develop colitis, indicating that BPI deletion within intestinal epithelial cells is not the cause of ulcerative colitis. This may be because the intestinal mucosa relies on a variety of antimicrobial proteins or peptides to maintain its stability (28).

Clinically, human BPI is related to ulcerative colitis (11–15). We used DSS as a model to induce acute experimental colitis in mice, as previously reported (15), in order to understand the role of BPI in the pathogenesis of ulcerative colitis. Our results showed that colonic lesions were found mainly on the mucosal surface, indicating that knockout of BPI promoted more severe damage to the colonic mucosa of the mice, which was consistent with the clinical hypothesis that BPI plays a protective role in the early stages of ulcerative colitis (12).

DSS is toxic to intestinal epithelial cells, which causes a decrease in the mucosal barrier, resulting in increased bacterial translocation. This causes blood neutrophils to infiltrate into the local infection site by chemotaxis *via* IL-8. These cells maintain the intestinal stability by phagocytosing and killing bacteria. Our results showed that BPI was expressed at sidewall and bottom of colon crypts in WT mice. In colitis model, the level of BPI was increased at bottom of colon crypts but decreased at the sidewall of crypt which may due to the mucosal injury. A BPI deficiency resulted in an increase of epithelial permeability of colon which led to LPS from colon into blood. This increased serum LPS stimulated the liver to synthesize and secrete increased levels of serum LBP. LPS permeabilized from colon also can induced an increase in the levels of various cytokines, such as TNF-α, IL-1β, IL-2, IL-3, and IFN-γ in the gut. The MPO results showed no significant changes between WT and KO mice, indicating that BPI deficiency might not affect neutrophils, which is the major resource of MPO. As reported before (29), BPI protein is closely associated with dendritic cell, and dendritic cell is a bridge between innate and adaptive immunity, which need to study in the future.

We did not detect any bacteria in the anti-coagulated blood and spleen homogenates of mice subjected to DSS. We then assessed the fecal flora of the mice. Our results showed that BPI knockout decreased the fecal flora diversity. The greatest differences in the number of bacteria were in unclassified and gram-negative bacteria, such as *Bacteroides.*

In summary, our study constructed BPI KO mice, which were then subjected to dextran sulfate sodium (DSS) colitis. The results showed that BPI KO developed worse colitis than control mice, as evidenced by increased colitis symptoms and colonic mucosal damage, elevated levels of serum LPS, and a disrupted microbiome. Thus, BPI may be a potential target for the treatment of ulcerative colitis (UC) in humans.

## Data Availability Statement

The 16s rDNA data has been uploaded to NCBI—BioProject ID: PRJNA670293.

## Ethics Statement

The animal study was reviewed and approved by the Animal Experiment and Experimental Animal Welfare Committee of Capital Medical University.

## Author Contributors

The study was designed by QK, YA, and JZ. Experiments were performed by QK, ZL, YK, and LL. Results were analyzed by QK and ZL. QK wrote the paper. All authors read and revised the paper. All authors contributed to the article and approved the submitted version.

## Funding

This work was supported by the Beijing Municipality Natural Science Foundation (#7172017).

## Conflict of Interest

The authors declare that the research was conducted in the absence of any commercial or financial relationships that could be construed as a potential conflict of interest.

## Supplementary Material

Supplementary Figure 1 RT-PCR results of BPI KO mouse testis were sequenced and compared with murine BPI mRNA. Using the BLAST function on the NCBI website, the sequencing results were compared with the mouse BPI mRNA. The 200 bp fragment (Query) overlaps with the tail of the 1^st^ exon (1–184) and the head of the 4^th^ exon (429–587), but the 2^nd^ and 3^rd^ exons were absent. Thus, the 2^nd^ and 3^rd^ exons of BPI were successfully knocked out of the mice.
