# Supplementary figures and images for: Bactericidal Permeability Increasing Protein Deficiency Aggravates Acute Colitis in Mice by Increasing the Serum Levels of Lipopolysaccharide
Source: Front Immunol. 2021 Jan 21;11:614169. doi: 10.3389/fimmu.2020.614169 (PMC7858664; doi:10.3389/fimmu.2020.614169)

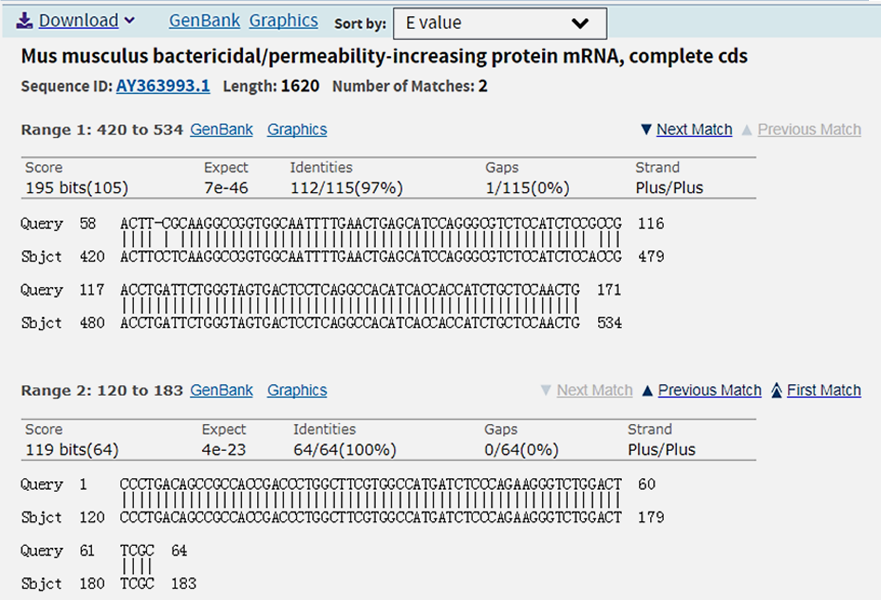

Supplement: Supplementary Figure 1 — RT-PCR results of BPI KO mouse testis were sequenced and compared with murine BPI mRNA. Using the BLAST function on the NCBI website, the sequencing results were compared with the mouse BPI mRNA. The 200 bp fragment (Query) overlaps with the tail of the 1st exon (1–184) and the head of the 4th exon (429–587), but the 2nd and 3rd exons were absent. Thus, the 2nd and 3rd exons of BPI were successfully knocked out of the mice. [file Image_1.tif]
